# The positive correlations of apolipoprotein E with disease activity and related cytokines in systemic lupus erythematosus

**DOI:** 10.1186/1746-1596-8-175

**Published:** 2013-10-21

**Authors:** Li-jun Song, Wei-wei Liu, Yu-chen Fan, Feng Qiu, Qi-lin Chen, Xing-fu Li, Feng Ding

**Affiliations:** 1 Department of Rheumatology, Qilu Hospital, Shandong University, 107# Wenhua Xi Road, Jinan 250012, P.R. China; 2 Department of Hepatology, Qilu Hospital, Shandong University, Jinan, P.R. China

**Keywords:** Systemic lupus erythematosus, Apolipoprotein E, Anti-inflammatory cytokine, SLEDAI

## Abstract

**Background:**

The aim of this study is to investigate the expression of apolipoprotein E (apoE) and the relationship between apoE and disease activity of SLE, and the possible effects of glucocorticoid on apoE and other cytokines activities in SLE patients.

**Methods:**

Forty treatment-naïve SLE patients and forty matched healthy controls were studied. All the SLE patients received prednisone 1 mg/kg/day for 28 consecutive days. The sera levels of apoE and related cytokines were evaluated by ELISA. The expression of apoE mRNA in peripheral blood mononuclear cells (PBMCs) was determined by real-time PCR.

**Results:**

Compared with healthy controls, the relative expression levels of ApoE proteins and sera levels were significantly up-regulated in active SLE patients. ApoE sera concentrations positively correlated with SLEDAI, anti-dsDNA antibody and the related cytokines including IL-6, IFN-γ and IL-10, and uncorrelated with the concentration of total cholesterol (TC) and triglyceride (TG) in SLE patients. After 4 weeks prednisone treatment, the relative mRNA expression of apoE and the serum levels of apoE and related cytokines decreased.

**Conclusions:**

ApoE correlated with disease activity and related cytokines in SLE patients. Glucocorticoid can down-regulate the expressions of apoE and related cytokines.

**Virtual slide:**

The virtual slide(s) for this article can be found here:http://www.diagnosticpathology.diagnomx.eu/vs/1646714011077325

## Introduction

Systemic lupus erythematosus (SLE) is a multisystem inflammatory and autoimmune disease. Despite the etiology of SLE has not been fully understood, the abnormal lymphocyte apoptosis, decreased clearance of activated T cells and involvement of multiple cytokines including IFN-γ [1], interleukin (IL)-10 [1] and IL-6 [2] have been demonstrated with the pathogenesis of SLE [3-5].

Apolipoprotein (apo) E is a multifunctional glycoprotein synthesized chiefly by the liver and the macrophage. It is implicated in human lipoprotein metabolism and cardiovascular disease [6]. Increasing studies have proved that apoE plays a key role in inhibiting the proliferation of T lymphocytes, regulating immune reactions and interacting with several cytokines [7-10]. Moreover, it has been suggested that apoE might play a pivotal role in modulating inflammatory and immune response in autoimmune diseases like multiple sclerosis (MS) and rheumatoid arthritis [11,12]. These lines of evidence indicate that apoE may play an important role in the pathogenesis of SLE.

Glucocorticoid remains the cornerstone of the treatment of SLE, despite advances in therapeutic protocols and development of new drugs [13]. GCs reduce the synthesis of pro-inflammatory cytokines, such as IL-6, tumor necrosis factor (TNF)-α [14] and anti-inflammatory cytokines such as IL-37 [15]. However, the effect of glucocorticoid on apoE remains unclear.

In this study, we compared the expression of apoE mRNA in peripheral blood mononuclear cells (PBMCs) and serum protein levels in SLE patients with healthy controls. In addition, we examined the disease activity using SLE disease activity index (SLEDAI) [16], anti-dsDNA antibody, IFN-γ, IL-6 and IL-10 in SLE to determine whether apoE is involved in the pathogenesis of SLE, and the possible effects of glucocorticoid on apoE and other cytokines activities in SLE patients.

## Materials and methods

### Subjects

Forty SLE patients (36 females and 4 males; range: 20 ~ 55 yrs) with systemic lupus erythematosus disease activity index (SLEDAI) ≥ 5 [16] were recruited into the present study. All patients who had visited the rheumatology ward of Qilu Hospital of Shandong University from November 2011 to October 2012 fulfilled the American College of Rheumatology (ACR) 1997 revised criteria for SLE [17]. Individuals with any other rheumatic diseases were excluded from the study. None of them had been treated with GCs or other immunosuppressive drugs prior to first collection of specimens. All of them received prednisone 1 mg/kg/day for 28 consecutive days. Forty sex- and age-matched healthy controls (36 females and 4 males; range: 21 ~ 57 yrs) were recruited into the present study, all of whom did not have any rheumatic conditions and dyslipidemia-related diseases. The study protocol was approved by the ethics committee of Qilu Hospital of Shandong University (No. 12126). All participants gave their informed consent for blood sampling.

### Blood samples

Peripheral venous blood was collected from each SLE patient and control subject. Samples were centrifuged at 3000 r/min for 5 minutes, and serum samples were stored at -80°C until use.

### Quantitative real-time polymerase chain reaction (RT -PCR)

Mononuclear cells were separated from heparinized blood with NycoPrep™1.077 (Axis-Shield, Norway) gradient centrifuge technique. Total RNA was extracted by Trizol Reagent (Invitrogen, America) according to instructions of the manufacturer. Approximately 1 μg of total RNA in 20 μg reactions was reversely transcribed to cDNA and 1.0 μg cDNA was used in the qRT-PCR proce.

Primer sequences used for the RT-PCR were as follows: ApoE, 5′- CTG CGT TGC TGG TCA CAT TC -3′ (forward), 5′- CTG GTG GGT TCT CCT TAT TG -3′ (reverse); and GAPDH, 5′- ACC ACA GTC CAT GCC ATC AC -3′ (forward), 5′- TCC ACC ACC CTG TTG CTG TA -3′ (reverse). Real-time PCR was performed using the SYBR Green I real-time PCR kit (TAKARA, Dalian, China) in an ABI PRISM 7300 Sequence Detector (Perkin-Elmer, Norwalk, CT, USA). The reaction was carried out for 40 cycles at 95°C for 5 s, 60°C for 45 s and 72°C for 45 s. Each sample was run in triplicate. The PCR products were separated in an agarose gel and to confirm the expected size in all cases. A melting-curve analysis was also performed to ensure specificity of the products. The relative expression level of ApoE was calculated with comparative threshold cycle (Ct) method and evaluated by:

$$\begin{array}{c} (2^{-\Delta \Delta \mathrm{Ct}},\Delta \Delta \mathrm{Ct}=\text{Patient}\;\left({\mathrm{Ct}}_{\text{ApoE}}-{\mathrm{Ct}}_{\text{GAPDH}}\right)\\ \;-\text{Mean}\;\text{of}\;\text{controls}\;\left({\mathrm{Ct}}_{\text{ApoE}}-{\mathrm{Ct}}_{\text{GAPDH}}\right) \end{array}$$

### Enzyme-linked immunosorbent assay (ELISA)

Determinations of serum apoE, IL-6, IFN-γ and IL-10 levels were quantified by ELISA, following the manufacturer’s instructions (Yonghui, Beijing, China). Detection of anti-dsDNA antibody was also quantified by ELISA which is becoming the most widely used method and has high sensitivity [18].

### Statistical analysis

Statistical analysis was performed using SPSS13.0. Data were expressed as mean ± SD when normally distributed and median ± IQR when non-normally distributed. All the data were analyzed with the non-parameter test. The comparisons among active patients, inactive patients and control group were performed by independent sample nonparametric tests. The correlations between apoE levels and SLEDAI, anti-dsDNA antibody, cytokines or serum lipid were analyzed by Spearman rank correlation. P < 0.05 was considered to be significant.

## Results

### Clinical characteristics of patients with SLE

The clinical and demographic features of SLE patients and healthy controls were shown in Table 1. The SLEDAI scores ranged from 11.45 ± 3.76 of pre-treatment to 7.90 ± 4.00 of post-treatment (p < 0.01). After prednisone treatment, the anti-dsDNA antibodies decreased significantly compared to the levels before treatment. The skin and mucosa lesion, hematological involvement, lupus nephritis, arthritis and serositis were the major manifestations in the present studied SLE patients.

**Table 1:** Clinical and demographic features of the studied subjects

**Characteristics**	**SLE patients**		**Healthy controls**
	**Pre-treatment**	**Post-treatment**	
No. of cases	40	40	40
Female, n (%)	36 (90.0%)	36 (90.0%)	36 (90.0%)
Male, n (%)	4 (10%)	4 (10%)	4 (10%)
Age, years (range)	33.7 (20 ~ 55)	33.7 (20 ~ 55)	34.8 (21 ~ 57)
Course of disease months	11.2 (1 ~ 26)	12.2 (2 ~ 27)	-
(range)			
SLEDAI (mean ± SD)	11.45 ± 3.76	7.90 ± 4.00	-
Skin and mucosa lesion	28 (70.0%)	15 (37.5%0)	-
Serositis, n (%)	15 (37.5%)	8 (20.0%)	-
Arthritis, n (%)	18 (45.0%)	4 (10.0%)	-
Lupus nephritis, n (%)	24 (60.0%)	20 (50%)	-
Hematologic involvement	25 (62.5%)	9 (22.5%)	-
NPSLE, n (%)	1 (2.5%)	0	-
ANA, n (%)	40 (100%)	39 (97.5%)	0
Anti-dsDNA antibody, (%)	18 (45.0%)	14 (35.0%)	0

### ApoE mRNA expression in SLE patients and controls

**Figure 1:**
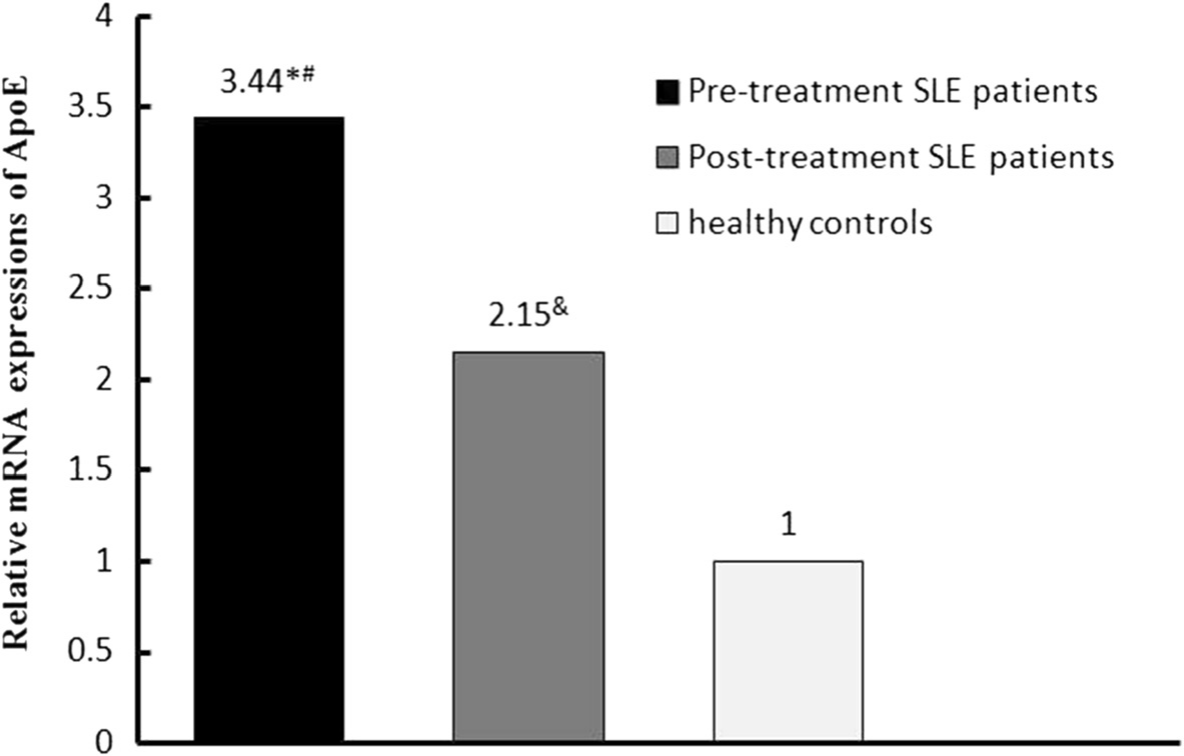
**Relative mRNA expressions of apoE in SLE patients (pre-treatment patients and post-treatment patients) and healthy controls.** Freshly isolated human PBMCs from SLE patients and healthy controls were quantified by RT-PCR. *, p < 0.05, Pre-treatment SLE patients vs. healthy controls; #, p < 0.05, Pre-treatment SLE patients vs. post-treatment SLE patients; &, p < 0.05, Post-treatment SLE patients vs. healthy controls.

Using the IQ5 software, the data were presented as the fold change in gene expression normalized to GAPDH. The relative expression of apoE mRNA in pre-treatment SLE patients increased by 3.44-fold compared with healthy controls (p < 0.05). After 4 weeks prednisone treatment, the relative expression of apoE mRNA decreased to 2.15-fold compared with healthy controls (p < 0.05) (Figure 1).

### Serum apoE, IL-6, IFN-γ and IL-10 levels in SLE patients and controls

As shown in Table 2, the plasma levels of all the tested cytokines including apoE, IL-6, IFN-γ and IL-10 in pre-treatment SLE patients were significantly up-regulated compared with healthy controls (p < 0.05). The levels of apoE, IL-6, IFN-γ and IL-10 were significantly decreased in the SLE patients after prednisone treatments.

**Table 2:** **The serum levers of apoE and other cytokines [pg/ml, M (Q**_**1**_, **Q**_**3**_**)]**

**Items**	**SLE patients**		**Controls**
	**Pre-treatment**	**Post-treatment**	
N	40	40	40
ApoE	18.31 (13.09, 45.37)*^#^	12.95 (8.03, 30.50)&	8.10 (5.94, 10.81)
IL-6	0.86 (0.70, 1.72)*^#^	0.72 (0.58, 1.18)	0.67 (0.51, 0.82)
IFN-	41.06 (33.25, 118.51)*^#^	33.85 (17.00, 56.08)	34.61 (29.12, 43.23)
IL-10	39.35 (31.94, 92.33)*^#^	33.85 (17.33, 58.80)	31.82 (23.46, 44.48)

Note: *, p < 0.05, SLE (pre-treatment) compared with normal controls; #, p < 0.05, SLE (pre-treatment) compared with SLE (post-treatment); &, p < 0.05, SLE (post-treatment) compared with normal controls.

### Correlation of serum apoE levels with disease activity and related cytokines in SLE patients and healthy controls

**Figure 2:**
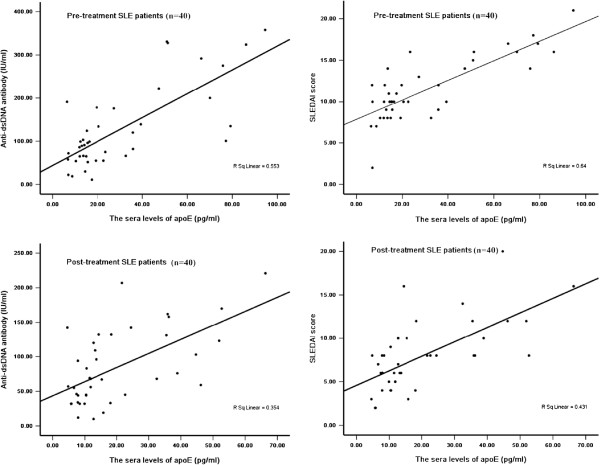
**Spearman rank correlation analysis of apoE with anti-dsDNA antibody and SLEDAI in SLE patients.** The sera apoE concentrations were positively correlated with anti-dsDNA antibody (r = 0.64, p < 0.01) and SLEDAI (r = 0.71, p < 0.01) in pre-treatment SLE patients. After prednisone treatment, the sera apoE concentrations were still positively correlated with anti-dsDNA antibody (r = 0.56, p < 0.01) and SLEDAI (r = 0.65, p < 0.01).

**Figure 3:**
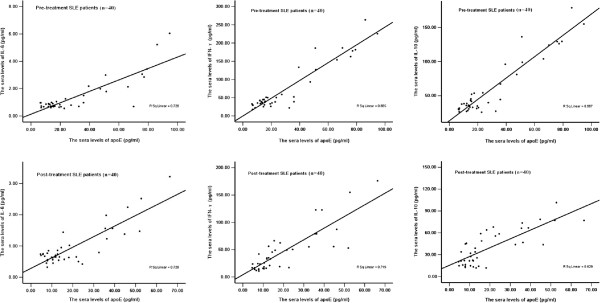
**Spearman rank correlation analysis of apoE with IL-6, IFN-γ and IL-10 in SLE patients.** The sera apoE concentrations positively correlated with IL-6 (r = 0.57, p < 0.01), IFN-γ (r = 0.78, p < 0.01) and IL-10 (r = 0.76, p < 0.01) in pre-treatment SLE patients. After prednisone treatment, the sera apoE concentrations still positively correlated with IL-6 (r = 0.54, p < 0.01), IFN-γ (r = 0.82, p < 0.01) and IL-10 (r = 0.70, p < 0.01).

ApoE levels revealed positive correlation with anti-dsDNA antibody in both pre-treatment SLE patients (r = 0.64, p < 0.01) and post-treatment patients (r = 0.56, p < 0.01) (Figure 2). Furthermore, a strong correlation was found between apoE sera levels and SLEDAI in pre-treatment patients (r = 0.71, p < 0.01) and post-treatment patients (r = 0.65, p < 0.01) (Figure 2). The sera apoE concentrations positively correlated with IL-6 (r = 0.57, p < 0.01), IFN-γ (r = 0.78, p < 0.01) and IL-10 (r = 0.76, p < 0.01) in pre-treatment SLE patients. After prednisone treatment, the sera apoE concentrations still positively correlated with IL-6 (r = 0.54, p < 0.01), IFN-γ (r = 0.82, p < 0.01) and IL-10 (r = 0.70, p < 0.01) (Figure 3). In healthy controls, the sera apoE concentrations also positively correlated with IL-6 (r = 0.87, p < 0.01), IFN-γ (r = 0.68, p < 0.01) and IL-10 (r = 0.72, p < 0.01).

### Correlations of ApoE with TC and TG in SLE patients

No statistically significant relationships between apoE and TC (r = 0.413, p = 0.435) or TG (r = 0.350, p = 0.724) were found in patients with SLE.

## Discussion

The results of the present studies show that apoE mRNA expression is increased in active SLE patients. The levels of apoE mRNA were low in stable SLE and healthy controls. In a similar way, the serum levels of IL-6, IFN-γ and IL-10 were also significantly increased in active SLE patients and low in stable SLE patients. These data indicate that apoE and related cytokines including IL-6, IFN-γ and IL-10 might take part in the pathogenesis of SLE.

ApoE is primarily synthesized in liver and brain astrocytes [19], however, it can also be produced by a wide variety of tissues including monocytes [20], adrenals and kidneys [21]. ApoE plays a vital role in modulating inflammation and oxidation since the function of apoE is closely linked with both pro-inflammatory and anti-inflammatory cytokines [6,10]. It has been reported that apoE-deficient (apoE^-/-^) macrophages ingest fewer apoptotic lymphocytes than wild type macrophages in animal experiment. What’s more, there is markedly T lymphocyte activating and attenuated initial immune response in apoE^-/-^ mice compared with wild type [22].

Several researches have confirmed that multiple cytokines such as IFN-γ [1,23], IL-10 [1,24] and IL-6 [2,24] take part in the pathogenesis of SLE by contributing to inflammatory and immunological responses. The significant increases of IFN-γ, IL-6 and decrease of IL-10 production in apoE^-/-^ mice have been shown in some report [8]. Inflammatory cytokine levels obviously decreased by injecting exogenous apoE [11]. Using IFN-γ to stimulate apoE^-/-^and apoE^+/+^ mice, the expressions of CD40, CD80 and the major histocompatibility complex class (MHC) II molecules on macrophages in apoE^-/-^ mice were increased compared with ApoE^+/+^[25]. This indicates that apoE at physiological level can affect antigen-presenting function and inhibit activation of T cells by down regulating the expression of MHCII and co-stimulatory molecules on antigen-presenting cells processed by IFN-γ. Taken together, apoE functions as anti-inflammatory cytokine in vivo and plays an important role in negative immune regulation.

The maintenance of immune homeostasis depends on the balance of pro-inflammatory cytokines and anti-inflammatory cytokines. The imbalance of immune system potentially results in the development of autoimmune disorders. It has been reported that multiple cytokines including pro-inflammatory such as IL-6 and IFN-γ [15] and anti-inflammatory cytokines such as IL-10 [26] and progranulin protein (PGRN) [27] and multiple immunocytes such as Treg [28], Th17 [29] and B cell [30] contribute to the pathogenesis of SLE. Our present study finds that apoE is elevated in active SLE patients accompanying the increase of IFN-γ, IL-6 and IL-10 and strongly correlated with IFN-γ, IL-6 and IL-10 serum levels. Based on the strong correlations between apoE and the above cytokines in SLE patients, we speculate that apoE may have an anti-inflammatory and immunoregulatory effect in SLE patients by interacting with other cytokines. The increase of apoE resulting from the increase of pro-inflammatory cytokines is a compensatory reaction; however, this compensation usually cannot fully antagonize the effect of pro-inflammatory cytokines, which partially lead to the development of SLE. The positive correlations between apoE and IFN-γ, IL-6 and IL-10 in healthy controls and active SLE indicate that both pro-inflammatory and anti-inflammatory cytokines keep the balance to the greatest extent in steady and active state.

In this study, our data clearly show that serum levels of apoE are closely correlated with SLEDAI scores as well as anti-dsDNA antibody levels. SLEDAI is the most widely accepted clinical model for SLE disease activity in most countries [31]. In addition, anti-dsDNA antibody plays a crucial role in the pathogenesis of SLE and is believed to be one of the evaluating factors of SLE disease [32]. It has been reported that both pro-inflammatory and anti-inflammatory cytokines increased accompanying the increase of SLEDAI score and anti-dsDNA antibody in active SLE [15,27]. SLEDAI score and anti-dsDNA antibody are closely related with SLE disease activity and apoE positively correlated with SLEDAI score and anti-dsDNA antibody, which means the aberrant expressions of apoE were related with SLE disease activity.

Glucocorticoid can inhibit Th1 cytokine production in T cells and potentially enhance Th2 cytokine synthesis in antigen-presenting cells [33-35]. Glucocorticoid binds to cytoplasmic glucocorticoid receptors and forms receptor-corticosteroid complex, which translocates to the nucleus and regulates the transcription of target genes [36]. The present study found that glucocorticoid can affect the serum levels of both pro-inflammatory and anti-inflammatory cytokines, which accords with our previous studies [15,27].

ApoE is mainly presented with the lipoprotein bound form, found in chylomicrons, very low density lipoproteins, and high density lipoproteins, as well as a key regulator of lipid and cholesterol metabolism [37,38]. In our present study, there are no correlations between apoE and TC or TG, which is maybe due to the polymorphism of apoE gene [39,40]. The relationship between polymorphisms of apoE gene and disease activity and related cytokines in SLE will be the key in our next study.

In conclusion, the present data demonstrate that apoE is up-regulated in active SLE patients. ApoE positively correlates with SLEDAI, anti-dsDNA antibody and related cytokines such as IL-6, IFN-γ and IL-10, which indicates that apoE is closely related with SLE disease activity and probable is involved in the pathogenesis of SLE.

## Competing interests

The authors declare that they have no competing interests.

## Authors’ contributions

LS and WL did the experiments and wrote the manuscript; FQ and QC did experiments. YF made contributions to analysis and interpretation of data. FD and XL designed the experiments and revised the manuscript. All authors read and approved the final manuscript.
